# Multi Dynamic Extraction: An Innovative Method to Obtain a Standardized Chemically and Biologically Reproducible Polyphenol Extract from Poplar-Type Propolis to Be Used for Its Anti-Infective Properties

**DOI:** 10.3390/ma12223746

**Published:** 2019-11-13

**Authors:** Vincenzo Zaccaria, Emanuele Ugo Garzarella, Carmen Di Giovanni, Fabio Galeotti, Lucia Gisone, Davide Campoccia, Nicola Volpi, Carla Renata Arciola, Maria Daglia

**Affiliations:** 1 Department of Drug Sciences, Medicinal Chemistry and Pharmaceutical Technology Section, Pavia University, Viale Taramelli 12, 27100 Pavia, Italy; vincenzo.zaccaria01@universitadipavia.it (V.Z.); giusymarialucia.gisone01@universitadipavia.it (L.G.); 2 Department of Pharmacy, Nutraceutical Lab, University of the Naples, Federico II, Via D. Montesano 49, 80131 Napoli, Italy; emanueleugo.garzarella@unina.it (E.U.G.); carmen.digiovanni@unina.it (C.D.G.); 3 Department of Life Sciences, University of Modena and Reggio Emilia, Via Campi 213/D, 41121 Modena, Italy; fabio.galeotti@unimore.it (F.G.); nicola.volpi@unimore.it (N.V.); 4 Laboratorio di Patologia delle Infezioni Associate all’Impianto, IRCCS Istituto Ortopedico Rizzoli, via di Barbiano 1/10, 40136 Bologna, Italy; 5 Department of Experimental, Diagnostic and Specialty Medicine, University of Bologna, via San Giacomo 14, 40126 Bologna, Italy; 6 International Research Center for Food Nutrition and Safety, Jiangsu University, Zhenjiang 212013, China

**Keywords:** multi dynamic extraction (M.E.D.) method, poplar-type propolis, standardized polyphenolic mixture, antimicrobial activity, antibiotic-resistant bacteria, anti-infective agent, anti-infective biomaterials

## Abstract

Antimicrobial activity is a well-known property of propolis, making it a candidate for antimicrobial surfaces in biomedical devices. Nevertheless, large-scale use of propolis as an anti-infective agent is limited by the heterogeneity of its chemical composition and consequent variation in antimicrobial activity. The aim of this study was to demonstrate that the multi dynamic extraction (M.E.D.) method produces standardized polyphenolic mixtures from poplar-type propolis, with reproducible chemical composition and anti-microbial activity, independently from the chemical composition of the starting raw propolis. Three raw propolis samples, from Europe, America, and Asia, were analyzed for their polyphenol chemical composition by means of HPLC–UV and then combined to obtain three mixtures of propolis, which werme submitted to the M.E.D. extraction method. The chemical composition and the antimicrobial activity of M.E.D. propolis against bacteria and fungi were determined. The three M.E.D. propolis showed similar chemical compositions and antimicrobial activities, exhibiting no relevant differences against antibiotic-susceptible and antibiotic-resistant strains. The batch-to-batch reproducibility of propolis extracts obtained with the M.E.D. method encourages the design of drugs alternative to traditional antibiotics and the development of anti-infective surface-modified biomaterials.

## 1. Introduction

Propolis is a natural resinous product, processed by bees from a range of plants for use in construction of their hives [1,2]. Its composition varies depending on its botanical and geographical origins [3], although the different types of propolis do have a shared chemical nature [4]. In fact, in addition to resin (50%), wax (30%), essential oils (10%), pollen (5%), and mineral salts (2%), the main components of propolis are polyphenols, including flavonoids, phenolic acids, and their esters [5].

The analysis of the chemical composition and biological properties of propolis, as well as its use in drugs, foods, and cosmetics requires an extraction process so as to remove impurities and inert materials while preserving the majority of the plant secondary metabolites, especially the polyphenol fraction [4,5,6]. Until now, this has been generally achieved by extraction with solvents, in particular with 70% ethanol. Hydro-alcoholic extraction results in wax-free tinctures containing variable amounts of bioactive compounds, including the phenolic substances [7,8]. Nevertheless, while simple and effective, this method suffers from some limits in application due to the potential presence of ethanol residues in the resultant pharmaceutical products, foods, and cosmetics.

Therefore, the production of non-ethanolic propolis extracts represents an important prospect, with progress focused on solubilization of the active molecules of propolis in aqueous or oily solvents, as phenolic compounds were found at 10-fold lower concentrations in these conditions compared to ethanolic extracts [9,10]. A new, patented propolis extraction method, called multi dynamic extraction (M.E.D.), has recently been proposed [11,12]. M.E.D. allows the preparation of non-ethanolic propolis extracts (M.E.D. extracts) with reliable polyphenolic contents, in contrast to the majority of other extraction methods that yield a poorly replicable chemical composition. On the contrary, M.E.D. extracts possess a standardized polyphenol composition containing 5–20% phenolic acids and 50–80% flavonoids (20–30% flavones and flavonols, 30–50% flavanones and dihydroflavonols, 5–20% glycosilated flavonoids and terpenoids), with these extracts being rich in six active compounds, i.e., galangin, chrysin, pinocembrin, apigenin, pinobanksin, and quercetin, with a relative concentration of about 40% (w/w) [13]. From its composition, M.E.D. extract can be defined as a poplar-type propolis polyphenolic mixture, rich in bioactive compounds, and highly purified from impurities and inert materials [12].

Propolis demonstrates many healthy properties, of which the most studied is antimicrobial activity [5,14,15,16,17], which has been described against multiple bacterial strains and fungi [18,19]. The complex mechanism by which propolis exerts its antimicrobial activity is yet to be elucidated and could involve synergisms between different polyphenols [20]. The major problems regarding these studies on the antimicrobial properties of propolis consist in the variation in composition of the studied propolis extracts, which give different results in terms of antimicrobial activity and make it impossible to compare results obtained from different propolis extracts. A further limit of the literature data is that the chemical composition of the tested extracts is different from that of the extracts currently used as drug, food, or cosmetics ingredients and so these results have limited practical applications. In summary, several factors such as origin, composition, and extraction method can influence the biological behavior of the product. On the basis of these considerations, following a request from the European Commission about a list of propolis health claims, the opinion of the European Food Safety Authority (EFSA) was that a cause–effect relationship cannot be established between the consumption of propolis and the claimed effects, due to the fact that “*type and content of flavonoids in propolis may vary depending on the specific propolis raw material as well as the extraction and preparation methods*”.

The aim of our study is to demonstrate the chemical and biological reproducibility of poplar-type propolis extracts obtained using a M.E.D. method on a combination of poplar-type propolis of different geographical origins. Thus, the chemical composition of nine hydroalcoholic propolis extracts and three non-ethanolic M.E.D. propolis was evaluated by high-performance liquid chromatography coupled with UV detection and mass spectrometry (HPLC–UV–MSn) and these were compared. In addition, the antimicrobial activity was used as a validation method of the extractive process M.E.D. to show the biological reproducibility of M.E.D. propolis.

## 2. Materials and Methods

### 2.1. Materials

HPLC-grade water was obtained from a LC-Pak™ Millex system (Millipore Corporation, Billerica, MA, USA). Formic acid, MS grade methanol, quercetin, apigenin, pinobaskin, crysin, pinocembrin, and galangin were obtained from PhytoLab, Vestenbergsgreuth, Germany.

### 2.2. Hydroalcoholic Propolis Extract Preparation

Three poplar-type raw propolis samples were obtained from as many European regions (Italy (E1), Spain (E2), and Turkey (E3)), three further poplar-type raw propolis samples were obtained from three different Southern American regions (Uruguay (sA1), Mexico (sA2), and Argentina (sA3)), and the last three poplar-type raw propolis samples were collected from distinct Asian regions (Mongolia (A1), Kazakhstan (A2), and north China (A3)). Samples (20 mg each) were dissolved in 2 mL of 70% ethanol. After vigorous mixing and sonication for 30 min, polyphenols were extracted at 70 °C in a water bath for 2 hours under continuous mixing. After centrifugation at 10,000 RPM for 10 min, hydroalcoholic propolis extracts were analyzed by RP-HPLC–PDA–ESI–MSn.

### 2.3. M.E.D. Propolis Preparation

In order to determine the chemical composition and test the antibacterial activity of M.E.D. propolis, three mixtures (mix A, mix B, and mix C) were prepared combining a European, an American, and an Asian poplar-type raw propolis sample (Eu + Am + As). M.E.D. propolis A, B, and C, respectively, were obtained from each raw mixture using M.E.D. (multi dynamic extraction), as reported in [11]. In brief, raw propolis mixtures were submitted to the M.E.D. extraction process, comprising several steps. These steps consisted of an initial aqueous extraction from dewaxed raw propolis, followed by a series of extractions on the residue using an ethanol/water mixture, with each extraction being carried out on the residue from the previous extraction using a higher percentage of ethanol. The combined extracts were mixed and concentrated by distillation to a residual humidity value ranging from 15 to 20% (w/w). The concentrated extracts were then analyzed by RP-HPLC–PDA–ESI–MSn and submitted to an antimicrobial assay.

### 2.4. Analyses of Hydroalcoholic Propolis Extracts and M.E.D. Propolis by RP-HPLC–PDA–ESI–MSn

The chromatographic analyses were performed by means of the RP-HPLC-PAD-ESI-MSn method, set up by Cui-ping et al., with some modifications [21]. These were performed using an Agilent 1100 VL series mass spectrometer (Agilent Technologies, Inc., Santa Clara, CA, USA), which was further used on-line with HPLC equipment. The electrospray interface was set in negative ionization mode with the capillary voltage at 3500 V and a temperature source of 350 °C in full scan spectra (200–2200 Da, 10 full scans/s). Nitrogen was used as a drying (9 L/min) and nebulizing gas (11 p.s.i.). Software versions were 4.0 LC/MSD trap control 4.2 and Data Analysis 2.2 (Agilent Technologies, Inc., Santa Clara, CA, USA). Compound separation was obtained with an analytical Synergi Fusion RP-18 column (150_4.6 mm, 5_m), equipped with a Hypersil Gold C18 precolumn (10 _2.1 mm, 5_m), all produced by Phenomenex (Torrance, CA, USA). The mobile phase used was acidified water, with 0.1% formic acid (eluent A) and methanol (eluent B). The run time was 110 min in total, including the reconditioning of the column. The flow rate was maintained at 1.00 mL/min, and the temperatures of the autosampler and column were kept at 4 and 33 °C. The volume of injection was set to 5 L. The elution method is described in Table 1. Chromatograms were registered at 260 nm. The HPLC-ESI-MSn data were collected using Xcalibur software (Xcalibur 2.0, Thermo Fisher Scientific, Waltham, MA, USA).

### 2.5. Antimicrobial Assays

In order to determine the inhibitory effects of M.E.D. propolis against different microorganisms, these experiments were carried out using the broth dilution method according to the procedures of the Clinical and Laboratory Standards Institute (CLSI), so as to determine the minimum inhibitory concentration (MIC), defined as the lowest concentration of an antimicrobial agent that can inhibit the growth of microorganisms.

Each dry M.E.D. propolis extract (A, B, or C) was resuspended in 50% (*v*/*v*) ethanol/water to obtain a final concentration of 50 mg/mL. A blank sample was also prepared, without adding any extract. Then, the three samples were serially diluted 1:2 in 50% ethanol, and 0.8 mL of each dilution were mixed with 7.2 mL of the specific agar culture medium, previously equilibrated at 70 °C, to finally cover a polyphenol concentration range between 0.007 mg/mL and 0.872 mg/mL. Once perfectly mixed by vortexing, agar culture medium was added to each propolis extract at different concentrations and then poured into a 6 mm Petri plate, and a cell suspension from a frozen vial was plated at about 5 × 10^3^ CFU/spot. As a positive control, some plates were prepared with the culture medium containing 0.8 mL of the blank stock. The growing media and conditions were chosen according to microorganism species (Table 2).

A large panel of bacterial and fungal species (Table 2) were used to test the antimicrobial activity of M.E.D. propolis, sourced from the American Type Culture Collection (ATCC) and clinical isolates (Code L) provided by Naicos srl, Milan, Italy (IRCCS Policlinico San Donato, San Donato Milanese, Italy; Centre Hospitalier Universitaire de Limoges, Limoges, France; International Health Management Associates, Inc., Shaumburg, IL, USA; Micromyx, LLC, Kalamazoo, MI, USA; Ospedale Busto Arstizio, Busto Arsizio, Italy; MM: IRCCS Multimedica, Milan, Itay; Rockville, MD, USA; S. Raffaele Hospital, Milan, Italy). The tested microorganisms included both drug-resistant species, showing resistance against one or more antibiotics, and sensitive species, i.e., non-resistant (Table 7). Tables 7 and 8 present the MIC values of selected antibiotics as reported by the literature where available, these were used as positive control.

### 2.6. Statistical Analysis

The values represent mean values of at least 3 replications. Data were analyzed by analysis of variance (ANOVA) with the statistical package GraphPad PRISM v 6.0 (2015) (GraphPad Software Inc., San Jose, CA, USA). Means were separated with the Tukey’s HSD method.

## 3. Results

### 3.1. RP-HPLC–PDA–ESI–MSn Analyses of Hydroalcoholic Propolis Extracts

Raw poplar-type propolis materials, obtained from three European regions (Eu1, Eu2, Eu3), three Southern American regions (Am1, Am2, Am3), and three Asian regions (As1, As2, As3) were submitted to hydroalcoholic extraction. The extracts were analyzed by means of RP-HPLC–PDA–ESI–MSn. The main flavonoid species, flavonols (galangin, quercetin), flavones (chrysin, apigenin), and flavonones (pinocembrin, pinobanksin), were identified on the basis of their UV and mass spectra, checking the molecular ion and fragment ions against the fragmentation patterns of standard molecules (Table 3) [12].

After the initial identification of quercetin, apigenin, pinobaskin, chrysin, pinocembrin, and galangin, they were determined by an on-line HPLC–UV according to methods previously described (Table 4).

The results (Table 4) showed that while the total polyphenol contents of the Asian hydroalcoholic propolis extracts were similar (mean value: 46.3% w/w, standard deviation: 0.8), the total polyphenol content of the American propolis samples ranged from 37.8 to 59.3 (mean value: 50.1%, standard deviation: 11.1), as was the case for the total polyphenol contents of the European propolis samples, which ranged from 38.2 to 42.6 (mean value: 40.6%, standard deviation 2.2).

**Figure 1:**
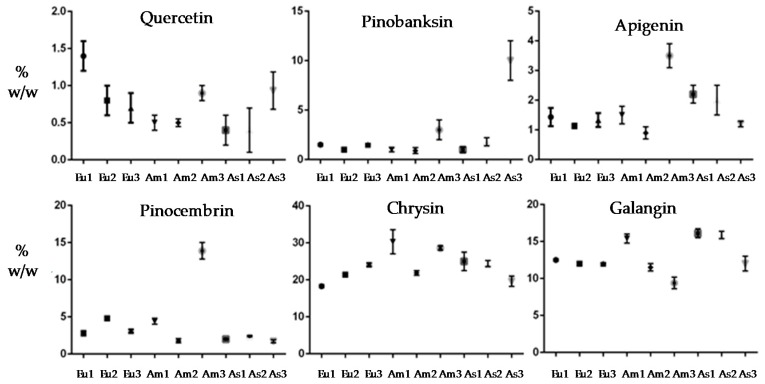
Analysis of Variance: contribution of geographical origin (country) on the relative percentage (% w/w) of each main flavonoid species: flavonols (galangin, quercetin), flavones (chrysin, apigenin), and flavonones (pinocembrin, pinobanksin).

As far as the relative percentage of each main flavonoid compound is concerned, the analysis of variance (ANOVA) was used to evaluate whether the concentration of each compound was statistically different between the hydroalcoholic propolis extracts, considering their different origins. The results, reported in Table 5, showed that the relative percentages of the polyphenols were often statistically different, confirming that the high variability of propolis raw materials of different origin leads to propolis extracts with different compositions when using common extraction methods (i.e., hydroalcoholic extraction) (Figure 1).

### 3.2. RP-HPLC–PDA–ESI–MSn Analyses of Non-Ethanolic M.E.D. Propolis

Raw propolis materials obtained from European (Eu1, Eu2, Eu3), Southern American (Am1, Am2, Am3), and Asian regions (As1, As2, As3) were combined to obtain three mixtures of European, American, and Asian poplar-type propolis (Eu + Am + As). Each mixture was submitted to the M.E.D. extraction process to give the extracts A, B, and C, respectively.

**Figure 2:**
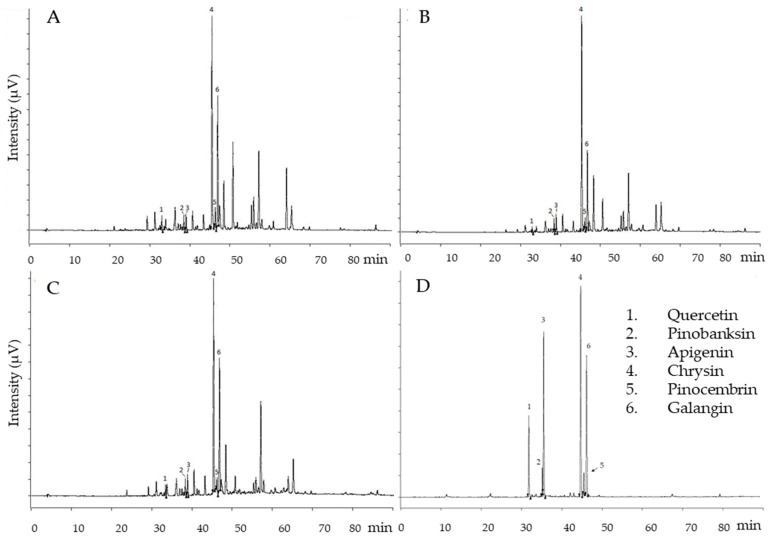
Chromatograms registered at λ 260 nm. (**A**) Multi dynamic extraction (M.E.D.) propolis extract A; (**B**) M.E.D. propolis extract B; (**C**) M.E.D. propolis extract C; (**D**) Standard compounds: 1—quercetin; 2—pinobainskin; 3—apigenin; 4—chrysin; 5—pinocembrin; 6—galangin.

**Figure 3:**
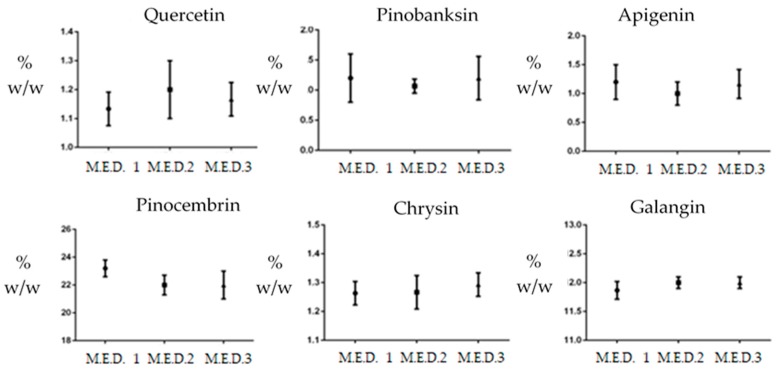
Analysis of Variance: contribution of M.E.D. extraction process on the relative percentage (% w/w) of each main flavonoid species: quercetin, pinobanksin, apigenin, chrysin, pinocembrin, and galangin.

After the identification of quercetin, apigenin, pinobaskin, chrysin, pinocembrin, and galangin on the basis of their UV and mass spectra (Table 3 and Figure 2D), they were quantified by means of HPLC–UV analyses (Table 6). The chromatograms acquired at λ 260 nm for each M.E.D. propolis extract are reported in Figure 2A–C. The relative percentages of each flavonoid were tested with ANOVA, showing that no differences were found in flavonoid composition between the non-ethanolic M.E.D. propolis extracts (Figure 3).

### 3.3. Antimicrobial Activity of M.E.D. Propolis Extracts

The antimicrobial activity of the three M.E.D. propolis extracts was first tested against microorganism strains representing the major families: Gram-positive or Gram-negative bacteria and fungi.

As expected, MIC values showed that the three M.E.D. propolis extracts exerted antimicrobial activity, confirming literature data on this property of propolis [18,19]. In particular, low MIC values (ranging between 20 and 156 μg/mL) were found against *Aspergillus niger*, *Streptococcus pneumonia* penicillin-susceptible, *Moraxella catarrhalis*, *Atopobium vaginae*, and *Neisseria gonorrhoeae*. Moderate activity was found against *Staphylococcus spp* and *Gardnerella vaginalis*, (MIC value = 312 μg/mL). Poor effects were registered on the growth of *Candida spp* and *Clostridium spp*, shown by MIC values above 1250 μg/mL. No activity could be detected against *Bacteroides fragilis* and *Lactobacillus spp* (Table 7).

The results obtained by our experiments gave comparable MIC values for each extract obtained using the M.E.D. method against the same microorganisms, despite the different geographical origins of the three samples.

Therefore, since the chemical composition and the antimicrobial activity values of the three M.E.D. propolis extracts were found to be comparable, we randomly selected extract A to investigate the antimicrobial activity more widely, including against antibiotic resistant microbial strains, as reported in Table 8. In this case, MIC values confirmed the antimicrobial activity of the extract against certain types of microorganisms, revealing the same effects of propolis on both sensitive and resistant species.

## 4. Discussion

Three sets of three propolis samples from Europe, America, and Asia were analyzed for their polyphenol chemical composition by means of RP-HPLC–PDA–ESI–MSn, and then each set was combined to obtain three mixtures of propolis that were submitted to the M.E.D. extraction method. The analysis of variance showed that the chemical compositions of M.E.D. propolis extracts were similar, whereas those extracted from the single propolis raw materials showed different compositions. The three M.E.D. propolis were found to be not only chemically but also biologically reproducible. In fact, these showed comparable antimicrobial activity against tested bacteria and fungi. Although the MIC values reported in the literature are dependent on many variables such as the geographical origin of the propolis, its chemical composition, method of extraction, and type of antimicrobial activity measured, many studies report major activity of propolis targeting Gram-positive bacteria. Our investigation on poplar-type propolis yielded evidence of a lowest MIC against Gram-positive bacteria, with good results against some Gram-negative bacteria and poor activities against fungi. Our data are in line with literature data. In fact, Muliet al. [22] described similar antibacterial activity of propolis extracts against *Pseudomonas aeruginosa*, *Salmonella typhi, Escherichia coli*, *S. aureus*, and *Bacillus subtilis*, with propolis sourced from three regions of Kenya and propolis extracts obtained using different concentrations of ethanol. In another study, *C. albicans* was found to be the most resistant strain and *S. aureus* the most sensitive to a Portuguese propolis [23].

In a fairly recent work on the antimicrobial activity of propolis on *S. aureus*, evaluated through the determination of the MIC, Pamplona-Zomenhan et al. (2011) [24] found some MIC values agreed with the results reported in literature, while yet others differed [21,25,26,27,28]. These differences, certainly due to the use of different extraction methods and to the variability of the bacterial strains, could also be dependent on different propolis samples, underlining the difficulty in studying the properties of propolis via extracts with a variable composition.

With the commonly used extraction methods (i.e., hydroalcoholic extraction), propolis extracts show poorly replicable chemical composition and biological properties due to the variability in the raw material. Nevertheless, in view of the uses for propolis extracts in drugs, foods, and cosmetics, it is important to have non-ethanolic propolis extracts with a standardized chemical composition and the guarantee of final products with the same biological properties. In our study, we are the first to report the antimicrobial activities of three mixtures of poplar-type propolis from different geographical areas with the same polyphenol content, obtained using the M.E.D. method as described in the paper by Zaccaria et al. [11]. The extracts show the presence of the main flavonoid species, flavonols (galangin, quercetin), flavones (chrysin, apigenin), and flavonones (pinocembrin, pinobanksin), that can be involved in different inhibitory mechanisms of microbial activity, such as inhibition of the mobility of the bacteria or peptidoglycan synthesis [29,30], pinocembrin-mediated inhibition of quorum sensing [31], and adhesion blocking by galangin [32]. Galangin and apigenin are present in M.E.D. propolis extracts with a relative average percentage w/w of 14.15% and 1.19%, respectively. In the literature, galangin showed a MIC = 32 µg/mL against different types of *S. aureus* (ATCC25293, N315, and Mu50), significantly suppressing bacterial growth at concentrations of 4, 8, and 16 μg/mL [33]. In another paper, apigenin and galangin were investigated against sensitive and antibiotic resistant strains of *S. aureus*, *Enterococcus faecalis, Enterococcus faecium*, *E. coli*, and *P. aeruginosa*. Galangin was shown to have a MIC ranging from 25 to 50 µg/mL against six strains of *S. aureus*, but negligible activity against other species, partially inhibiting the growth of all six enterococci. Apigenin only displayed marginal activity against *S. aureus* and did not show any activity against enterocci [34]. Quercetin is present in M.E.D. propolis extracts at an average percentage w/w of 1.11%. A recent study revealed antibacterial activity against *S. aureus*, *E. coli*, *P. vulgaris*, *Shigella*, *P. aeruginosa*, and *Lactobacillus casei* var. shirota. *S. aureus* and *P. aeruginosa* were inhibited, with an MIC value = 20 µg/mL, while moderate activity was seen against *E. coli* and *P. vulgaris* (MIC = 30 µg/mL) and no activity against *Shigella flexneri* and *Lactobacillus casei* with MIC > 300 µg/mL [35]. Other compounds, pynocembrin, and pinobanksin, revealed weak antibacterial activities against Gram-positive and Gram-negative strains. Pynocembrin, present at a 1.30% average percentage in M.E.D. propolis extract, did not inhibit the growth of *S. aureus*, as described in a paper by Soromou et al. [36]. Pinobanskin (1.20% average percentage in M.E.D. propolis extracts) showed MIC > 515 µg/mL against four strains of *S. aureus*, but no data were reported against *E. coli*. [37]. Finally, chrysin showed MIC > 50 µg/mL against *S. aureus* ATCC25923 methicillin-sensitive, *S. aureus* ATCC43300 methicillin-resistant, and different strains of *E. coli* [38]. The comparison between MIC values reported in the literature for each flavonoid and the concentrations of these compounds present in M.E.D. propolis extracts, suggests that the antimicrobial activity of M.E.D. propolis is not purely due to these flavonoids, but is influenced in a more complex matrix effect. With this view, previous papers have reported that the antimicrobial activity of propolis can be ascribed to the synergistic effects of phenolics and flavonoids [14,39] and other compounds such as diterpenic acids [40].

Our study also evidenced interesting activities of M.E.D. propolis extracts against antibiotic resistant bacteria such as *Streptococcus pneumoniae* clindamycin/erythromycin resistant and to a lesser extent against different strains of *Staphylococcus*. Antibiotic resistance is a serious and urgent problem for public health. As published by the CDC web site, at least 2 million people are infected with antibiotic-resistant bacteria each year in the U.S., and at least 23,000 people die as a result. Infections caused by antibiotic-resistant microorganisms are very difficult to treat and, in most cases, require hospitalization and expensive therapeutic alternatives. The Antibiotic Resistant Gene Database (ARDB) evidences about 20,000 genes potentially able to mutate and acquire antibiotic resistance by multiple mechanisms of action [41]. Our results are encouraging and useful for the development of new antimicrobial agents in the near future, targeting multi-resistant bacteria strains in support of ongoing therapies. Our results also encourage the design of new anti-infective biomaterials alternative to antibiotic-loaded materials [42]. Since the 1990s, the search for biomaterials able to thwart bacterial contamination and infection has been exponential in terms of surface modifications and coatings [43,44,45]. Recently, this research has been focusing on making the material surfaces not only bactericidal but also functionalized with other favorable properties [46,47]. It is expected that the new generation of anti-infective biomaterials will be able to integrate with host tissues, exert anti-inflammatory activities, and promote wound healing, in addition to counteracting bacterial colonization [48]. Propolis is a bactericidal compound worthy of recognition as a candidate for preparing new anti-infective materials. It meets the expectations and corresponds with the newly emerging concepts of biocompatibility, being endowed with multifaceted therapeutic bioactivities, mainly attributable to its polyphenol content [1,5]. In a very recent study, cornstarch incorporated with propolis and hyaluronic acid was found to improve wound dressing [49]. Films based on polysaccharides loaded with propolis and vitamin C have been shown to be an effective bio-coating to accelerate the healing of diabetic wounds [50]. Polyvinyl alcohol (PVA) hydrogels loaded with a Brazilian propolis have been shown to be effective in promoting burn wound healing [51]. Recent advances in therapeutic strategies to control the immune response to implants have highlighted the role of propolis in modulating the behavior of neutrophils and in harmonizing the cellular responses to biomaterials. Indeed, propolis seems to be able to favor the transition of macrophages from the M1 to M2 phenotype, extinguishing inflammation [51]. A safe and long-term use of polymeric materials requires the application of anti-degrading agents with a wide range of actions. Raw propolis originating from two geographic regions of Europe was used to protect natural rubber materials from degradation by oxygen, ozone, and microorganisms [52]. The addition of propolis to a rubber mixture made the material resistant to thermo-oxidative aging and ozone and protected it from biodegradation [53]. As for the new and interesting field of nanomaterials, chitosan–propolis nanoparticles, prepared using propolis from Asia, exhibited the abilities to alter the zeta potential of *S. epidermidis*, inhibit biofilm formation by modulating gene expression, and synergize with antibiotics [54]. Propolis/polyurethane composite nanofibers and electrospun materials have been proposed for biomedical applications [55]. Propolis polyphenols, although beneficially bioactive molecules, pose problems of low bioavailability, and thus may benefit from targeted nanodelivery systems via nanocarriers [56]. In particular, permeation through the buccal mucosa appears to be a promising approach to bypass liver metabolism and release propolis-based nano-formulations and niosomes locally or systemically [57,58].

As far as the in vitro cytotoxicity, it has been reported in the literature that propolis extracts express toxic effects on tumor cells (the half-maximal inhibitory concentration IC50 sometimes approaching values as low as 2 µg/mL) and, to a lesser extent, even on normal cells (IC50: 58 µg/mL) [59,60]. In the early stages of the study of new biomaterials, investigations are required to assess their cytocompatibility under conditions close to the final clinical application. The propolis extracts here presented will be used as nanostructured coatings or nanoformulated drugs. Nanocoatings and nanoformulations can be expected to amplify the antibacterial properties while minimizing the side effects towards host cells.

In conclusion, this study suggests the use of a non-ethanolic mixture of poplar-type propolis with a standardized polyphenol content obtained by an extraction method such as M.E.D., so as to produce comparable activity data. Moreover, the analysis of the antimicrobial data suggests new potential medical applications for M.E.D. poplar-type propolis against antibiotic resistant microbial strains that will need to be further investigated in the future.

## Figures and Tables

**Table 1:** RP-HPLC–PDA–ESI–MSn analysis elution method.

Time (min)	% Eluent A	% Eluent B
0	85	15
30	60	40
65	45	55
70	38	62
85	0	100
90	0	100
100	85	15
110	85	15

**Table 2:** The growing conditions for the microorganisms selected to test propolis antimicrobial activity.

Microbial Strain	Media	Conditions
*S. aureus* methicillin-sensitive ATCC25923 (MSSA)(L1280) *S. aureus* methicillin-resistant (MRSA) (L4064) *S. aureus* MSSA + glycopeptide-interMediate resistant (GISA) (L3797) *S. aureus* MRSA + GISA (L3798) *S. aureus* clinda-inducible erm(A)^+^ (ND053410) *S. aureus* community acquired USA300 MRSA (ND054910) *S. aureus* MRSA + macrolides-resistant (ND060411) *S. hominis* ATCC27844 (L323) *S. epidermidis* (L147; ND052110; ND051710) *S. epidermidis* teicoplanin-resistant (ND042409) *S. capitis* MRSA (ND021008) *S. xylosus* MRSA (ND026108) *S. simulans* (ND029808) *S. haemolyticus* MRSA (L1730, ND040809; L1729) *E. coli* hyperpermeable (G1640) *E. coli* ATCC25922 (L1281) *E. coli* hyperpermeable (L4242; L47) *P. aeruginosa* ATCC27853 (L1367) *M. catarrhalis* (L3292) *A. baumannii* (L3030) *L. monocytogenes* ATCC13932 (L1450) *B. cereus* ATCC10702 (L85)	Mueller Hinton Agar	Aerobic, 24 h, 37 °C
*S. pneumoniae* penicillin-susceptible (L44) *S. pneumoniae* penicillin-resistant (L3917) *S. pneumoniae* clindamycin and erythromycin resistant (L1542) *S. pneumoniae* macrolide and erythromycin resistant (L1402)	Todd Hewitt Agar	Aerobic, 24 h, 37 °C
*C. parapsilosis* ATCC90018 (L3022) *C. parapsilosis* ATCC22019 (L4119) *C. albicans* ATCC24443 (L4120) *C. albicans* ATCC90028 (L3023) *C. guillermondii* ATCC6260 (L2065) *C. kruzei* (L2880) *A. niger* ATCC10535 (L53)	Sabouraud Dextrose Agar	Aerobic, 48 h, 37 °C
*G. vaginalis* (L1629; L1622; L1630) *A. vaginae* (ND736; ND737) *B. fragilis* ATCC25285 (L1011) *L. paracasei* (L1693) *L. plantarum* (L19) *L. gasseri* (ND787) *L. acidophilus* (ND786) *C. difficile* (L1365; L1366) *C. difficile* ATCC17858 (L4013)	Brucella Agar with 5% laked horse blood and 1% hemin and vitamin K	Anaerobic, 72 h, 37 °C
*N. gonorrhoeae* (L1600; L1601; L1599)	Brucella Agar with 5% laked horse blood, 1% hemin and vitamin K and 1% isovitalex	Anaerobic, 72 h, 37 °C
*C. perfrigens* (L4053) *C. perfrigens* ATCC13124 (L3697) *P. acnes* ATCC25746 (L1016)	Brucella Agar with 5% laked horse blood and 1% hemin and vitamin K	Anaerobic, 48 h, 37 °C

**Table 3:** Chromatographic and spectral properties of polyphenol compounds detected in propolis samples.

Peak number	RT (min)	UV absorption (λ_max_)	*m*/*z* [M-H]	Fragments (*m*/*z*)	Proposed Structure
1	31.8	256	301	151, 179, 257, 273	Quercetin
2	35.2	325	271	151, 165, 225, 253	Pinobaskin
3	35.5	267, 338	269	117, 149, 225	Apigenin
4	44.7	270	253	209	Chrysin
5	45.5	290	255	151, 187, 213	Pinocembri
6	46.2	261, 351	269	227	Galangin

**Table 4:** Relative percentage (% w/w) of the specific polyphenols occurring in the nine hydroalcoholic propolis extracts of different geographical origins, determined by HPLC-UV.

Polyphenols	Eu1	Eu2	Eu3
1-Quercetin	1.4 ± 0.6	0.8 ± 0.2	0.7 ± 0.4
2-Pinobanksin	1.5 ± 0.1	1.0 ± 0.2	1.5 ± 0.3
3-Apigenin	1.6 ± 0.3	1.1 ± 0.3	1.2 ± 0.3
4-Chrysin	18.3 ± 0.3	21.4 ± 0.2	24.1 ± 0.4
5-Pinocembrin	2.8 ± 0.3	4.8 ± 0.1	3.1 ± 0.2
6-Galangin	12.6 ± 0.1	12.0 ± 0.1	12.0 ± 0.2
Sum of percentages	38.2	41.1	42.6
-	**Am1**	**Am2**	**Am3**
1-Quercetin	0.5 ± 0-6	0.5 ± 0.1	0.9 ± 0.5
2-Pinobanksin	1.0 ± 0.2	0.9 ± 0.2	3.0 ± 0.8
3-Apigenin	1.5 ± 0.9	0.9 ± 0.3	3.5 ± 1.2
4-Chrysin	30.3 ± 3.3	22.2 ± 1.1	28.6 ± 0.6
5-Pinocembrin	4.4 ± 0.4	1.8 ± 0.3	13.9 ± 1.1
6-Galangin	15.4 ± 0.6	11.5 ± 0.4	9.4 ± 1.3
Sum of percentages	53.1	37.8	59.3
-	**As1**	**As2**	**As3**
1-Quercetin	0.4 ± 0.4	0.4 ± 0.5	0.9 ± 0.4
2-Pinobanksin	1.0 ± 0.2	1.8 ± 0.4	10.0 ± 2.0
3-Apigenin	2.2 ± 0.8	2.0 ± 1.7	1.2 ± 0.1
4-Chrysin	25.0 ± 2.5	24.4 ± 0.8	19.6 ± 1.4
5-Pinocembrin	2.0 ± 0.1	2.4 ± 0.1	1.7 ± 0.2
6-Galangin	16.1 ± 0.6	15.9 ± 0.5	12.0 ± 1.0
Sum of percentages	46.7	46.9	45.4

**Table 5:** Analysis of Variance: contribution of geographical origin (country) on the relative percentage (% w/w) of each main flavonoid species: flavonols (galangin, quercetin), flavones (chrysin, apigenin), and flavonones (pinocembrin, pinobanksin).

Comparisons	Significance					
	Quercetin	Pinobanksin	Apigenin	Chrysin	Pinocembrin	Galangin
EU 1 vs EU 2	Yes *	No **	No	No	Yes	No
EU 1 vs EU 3	Yes	No	No	Yes	No	No
EU 1 vs AM 1	Yes	No	No	Yes	Yes	Yes
EU 1 vs AM 2	Yes	No	No	No	No	No
EU 1 vs AM 3	No	No	Yes	Yes	Yes	Yes
EU 1 vs AS 1	Yes	No	No	Yes	No	Yes
EU 1 vs AS 2	Yes	No	No	Yes	No	Yes
EU 1 vs AS 3	No	Yes	No	No	No	No
EU 2 vs EU 3	No	No	No	No	Yes	No
EU 2 vs AM 1	No	No	No	Yes	No	Yes
EU 2 vs AM 2	No	No	No	No	Yes	No
EU 2 vs AM 3	No	No	Yes	Yes	Yes	Yes
EU 2 vs AS 1	No	No	Yes	No	Yes	Yes
EU 2 vs AS 2	No	No	Yes	No	Yes	Yes
EU 2 vs AS 3	No	Yes	No	No	Yes	No
EU 3 vs AM 1	No	No	No	Yes	Yes	Yes
EU 3 vs AM 2	No	No	No	No	Yes	No
EU 3 vs AM 3	No	No	Yes	Yes	Yes	Yes
EU 3 vs AS 1	No	No	Yes	No	No	Yes
EU 3 vs AS 2	No	No	No	No	No	Yes
EU 3 vs AS 3	No	Yes	No	Yes	Yes	No
AM 1 vs AM 2	No	No	No	Yes	Yes	Yes
AM 1 vs AM 3	No	No	Yes	No	Yes	Yes
AM 1 vs AS 1	No	No	No	Yes	Yes	No
AM 1 vs AS 2	No	No	No	Yes	Yes	No
AM 1 vs AS 3	No	Yes	No	Yes	Yes	Yes
AM 2 vs AM 3	No	No	Yes	Yes	Yes	Yes
AM 2 vs AS 1	No	No	Yes	No	No	Yes
AM 2 vs AS 2	No	No	Yes	No	No	Yes
AM 2 vs AS 3	No	Yes	No	No	No	No
AM 3 vs AS 1	No	No	Yes	No	Yes	Yes
AM 3 vs AS 2	No	No	Yes	No	Yes	Yes
AM 3 vs AS 3	No	Yes	Yes	Yes	Yes	Yes
AS 1 vs AS 2	No	No	No	No	No	No
AS 1 vs AS 3	No	Yes	Yes	Yes	No	Yes
AS 2 vs AS 3	No	Yes	No	Yes	No	Yes

* Yes means statistically significant difference between the relative percentage (% w/w) of each flavonoid in hydroalcoholic propolis extracts from different origins. ** No means no statistically significant difference between the relative percentage (% w/w) of each flavonoid in hydroalcoholic propolis extracts from different origins.

**Table 6:** Relative percentage (% w/w) of the specific polyphenols occurring in the three propolis extracts determined by high-performance liquid chromatography–UV (HPLC–UV).

Polyphenols	M.E.D. Propolis A	M.E.D. Propolis B	M.E.D. Propolis C
1-Quercetin	1.1 ± 0.05	1.2 ± 0.10	0.9 ± 0.06
2-Pinobanksin	1.2 ± 0.40	0.8 ± 0.11	1.6 ± 0.36
3-Apigenin	1.2 ± 0.30	1.0 ± 0.20	1.4 ± 0.04
4-Chrysin	23.2 ± 0.60	22.0 ± 0.71	22.0 ± 1.02
5-Pinocembrin	1.17 ± 0.04	1.4 ± 0.06	1.4 ± 0.04
6-Galangin	13.4 ± 0.15	14.7 ± 0.11	14.3 ± 0.10

**Table 7:** Minimum inhibitory concentration (MIC) values of the M.E.D. propolis extracts A, B, and C against the tested microbial strains, and of the antimicrobial drugs against their susceptible microrganisms.

Microbial Strain	MIC (µg/mL)				MIC (µg/mL)
	CODE	A	B	C	Antimicrobial agent
*Staphylococcus aureus* MSSA ATCC25923	L1280	312	312	312	-
*Staphylococcus epidermidis* ATCC12228	L147	312	312	312	-
*Escherichia coli hyperpermeable*	G1640	312	625	625	0.078, trimethroprim
*Moraxella catarrhalis*	L3292	39	78	78	0.3, ampicillin
*Streptococcus pneumoniae* penicillin-susceptible	L44	20	39	39	2.0, ampicillin
*Candida albicans* ATCC24443	L4120	1250	1250	1250	0.75, fluconazole
*Candida albicans* ATCC90028	L3023	1250	2500	2500	1.0, fluconazole
*Candida parapsilosis* ATCC90018	L3022	2500	2500	2500	4.0, fluconazole
*Candida kruzei*	L2280	2500	2500	2500	10.0, fluconazole
*Aspergillus niger* ATCC10535	L53	78	156	156	1000, fluconazole
*Bacteroides fragilis* ATCC25285	L1011	5000	>5000	>5000	6.0, cefoxitin
*Propionebacterium acnes* ATCC25746	L1016	>5000	>5000	>5000	1.8, clindamycin
*Clostridium difficile*	L1365	2500	2500	2500	0.6, vancomycin
*Clostridium difficile* ATCC17858	L4013	5000	2500	2500	1.6, vancomycin
*Atopobium vaginae*	ND736	156	156	156	0.478, ampicillin
*Lactobacillus gasseri*	ND787	5000	>5000	>5000	0.25, ampicillin
*Lactobacillus acidophilus*	ND786	>5000	>5000	>5000	1.0, clindamycin
*Neisseria gonorrhoeae*	L1600	156	156	156	16.0, ampicillin
*Neisseria gonorrhoeae*	L1601	156	78	78	-
*Gardnerella vaginalis*	L1629	312	312	156	0.020 ampicillin
*Gardnerella vaginalis*	L1630	312	312	312	-

**Table 8:** MIC values of M.E.D. propolis extract A and antimicrobial drugs on a wider microbial set.

Microbial Strain	CODE	Propolis Extract A MIC (µg/mL)	Antimicrobial Agent MIC (µg/mL)
*Escherichia coli*	L4242	312	0.12, ampicillin
*Staphylococcus aureus* GISA MSSA	L3797	625	-
*Staphylococcus aureus* GISA MRSA	L3798	312	-
*Staphylococcus haemolyticus* MRSA	L1730	312	-
*Staphylococcus hominis* ATCC27844	L323	625	0.046, ampicillin
*Staphylococcus capitis* MRSA	ND021008	156	-
*Staphylococcus xylosus* MRSA	ND026108	625	-
*Staphylococcus simulans*	ND029808	1250	-
*Staphylococcus haemolyticus* MRSA	ND040809	625	-
*Staphylococcus haemolyticus* MRSA	L1729	1250	-
*Staphylococcus aureus* Clinda-inducible erm(A)+	ND053410	625	64.0, ampicillin
*Staphylococcus aureus* Community Acquired USA300 MRSA	ND054910	625	-
*Staphylococcus aureus* MRSA macrolide-resistant	ND060411	625	-
*Staphylococcus aureus* MRSA	L4064	625	-
*Staphylococcus epidermidis* teicoplanin-resistant	ND042409	625	-
*Staphylococcus epidermidis*	ND052110	312	-
*Staphylococcus epidermidis*	ND051710	625	-
*Streptococcus pneumonia* clindamycin/erythromycin resistant	L1542	39	-
*Streptococcus pneumonia* macrolide/erythromycin resistant	L1402	39	-
*Streptococcus pneumoniae* penicillin-resistant	L3917	20	-
*Candida guillermondii* ATCC 6260	L2065	2500	2.5, fluconazole
*Candida parapsilosis* ATCC22019	L4119	1250	4.0, fluconazole
*Escherichia coli* ATCC25922	L1281	5000	5.0, ampicillin
*Escherichia coli*	L47	5000	0.12, ampicillin
*Pseudomonas aeruginosa* ATCC27853	L1367	5000	128.0, ampicillin
*Acinetobacter baumannii*	L3030	5000	4.0, ciprofloxacin
*Clostridium difficile*	L1366	5000	1.6, vancomycin
*Atopobium vaginae*	ND737	156	0.478, ampicillin
*Lactobacillus paracasei*	L1693	5000	0.12, penicillin
*Lactobacillus plantarum*	L19	5000	0.5, amoxicillin
*Neisseria gonorrhoeae*	L1599	156	16.0, ampicillin
*Gardnerella vaginalis*	L1622	312	0.020, ampicillin
*Listeria monocytogenes* ATCC13932	L1450	1250	0.563, ampicillin
*Bacillus cereus* ATCC10702	L85	312	2.0, penicillin
*Clostridium perfringens* HSR	L4053	5000	4.06, clindamycin
*Clostridium perfringens* ATCC13124	L3697	2500	0.188, clindamycin

## References

[1] Pasupuleti V.R., Sammugam L., Ramesh N., Gan S.H. (2017). Honey, propolis, and royal jelly: A comprehensive review of their biological actions and health benefits. Oxid. Med. Cell. Longev..

[2] Ghisalberti E.L. (1979). Propolis: A review. Bee World.

[3] Bankova V., Bertelli D., Borba R., Conti B.J., da Silva Cunha I.B., Danert C., Eberlin M.N., Falcão S.I., Isla M.I., Papotti G. (2016). Standard methods for *Apis mellifera* propolis research. J. Apic. Res..

[4] Gómez-Caravaca A.M., Gómez-Romero M., Arráez-Román D., Segura-Carretero A., Fernández-Gutiérrez A. (2006). Advances in the analysis of phenolic compounds in products derived from bees. J. Pharm. Biomed. Anal..

[5] Zabaiou N., Fouache A., Trousson A., Baron S., Zellagui A., Lahouel M., Lobaccaro J.A. (2017). Biological properties of propolis extracts: Something new from an ancient product. Chem. Phys. Lipids.

[6] Bankova V., Popova M., Trusheva B. (2014). Propolis volatile compounds: Chemical diversity and biological activity: A review. Chem. Cent. J..

[7] Woisky R.G., Salatino A. (1998). Analysis of propolis: Some parameters and procedures for chemical quality control. J. Apic. Res..

[8] Pietta P.G., Gardana C., Pietta A.M. (2002). Analytical methods for quality control of propolis. Fitoterapia.

[9] Kubiliene L., Laugaliene V., Pavilonis A., Maruska A., Majiene D., Barcauskaite K., Kubilius R., Kasparaviciene G., Savickas A. (2015). Alternative preparation of propolis extracts: Comparison of their composition and biological activities. BMC Complement. Altern. Med..

[10] Mello B.C.B.S., Petrus J.C.C., Hubinger M.D. (2010). Concentration of flavonoids and phenolic compounds in aqueous and ethanolic propolis extracts through nanofiltration. J. Food Eng..

[11] Zaccaria V., Curti V., Di Lorenzo A., Baldi A., Maccario C., Sommatis S., Mocchi R., Daglia M. (2017). Effect of green and brown propolis extracts on the expression levels of microRNAs, mRNAs and proteins, related to oxidative stress and inflammation. Nutrients.

[12] Galeotti F., Maccari F., Fachini A., Volpi N. (2018). Chemical composition and antioxidant activity of propolis prepared in different forms and in different solvents useful for finished products. Foods.

[13] Volpi N., Fachini A. (2017). Procedimento Per L’ottenimento di Estratti Integrali di Propoli Ricchi in Polifenoli e Dot, ati di Attività Antibatterica e Sua Applicazione Nella Prevenzione e Trattamento di Processi Infettivi di Origine Batterica.

[14] Castaldo S., Capasso F. (2002). Propolis, an old remedy used in modern Medicine. Fitoterapia.

[15] Kocot J., Kiełczykowska M., Luchowska-Kocot D., Kurzepa J., Musik I. (2018). Antioxidant potential of propolis, bee pollen, and royal jelly: Possible medical application. Oxid. Med. Cell. Longev..

[16] Havsteen B.H. (2002). The biochemistry and medical significance of the flavonoids. Pharmacol. Ther..

[17] Bankova V. (2005). Recent trends and important developments in propolis research. Evid. Based. Complement. Alternat. Med..

[18] Chee H.Y. (2002). In vitro evaluation of the antifungal activity of propolis extract on *Cryptococcus neoformans* and *Candida albicans*. Mycobiology.

[19] Ota C., Unterkircher C., Fantinato V., Shimizu M.T. (2001). Antifungal activity of propolis on different species of Candida. Mycoses.

[20] Kujumgiev A., Tsvetkova I., Serkedjieva Y., Bankova V., Christov R., Popov S. (1999). Antibacterial, antifungal and antiviral activity of propolis of different geographic origin. J. Ethnopharmacol..

[21] Miorin P.L., Levy Junior N.C., Custodio A.R., Bretz W.A., Marcucci M.C. (2003). Antibacterial activity of honey and propolis from *Apis mellifera* and *Tetragonisca angustula* against *Staphylococcus aureus*. J. Appl. Microbiol..

[22] EFSA Panel on Dietetic Products, Nutrition and Allergies (NDA) (2010). Scientific opinion on the substantiation of health claims related to propolis (ID **1242**, *1245*, 1246, **1247**, *1248*, 3184) and flavonoids in propolis (ID **1244**, *1644*, 1645, **3526**, *3527*, 3798, 3799) pursuant to Article 13(1) of Regulation (EC) No 1924/2006. EFSA J..

[23] Muli E.M., Maingi J.M. (2007). Antibacterial activity of *Apis mellifera* L. propolis collected in three regions of Kenya. J. Venom. Anim. Toxins Trop. Dis..

[24] Silva J., Rodrigues S., Feás X., Estevinho L. (2012). Antimicrobial activity, phenolic profile and role in the inflammation of propolis. Food Chem. Toxicol..

[25] Pamplona-Zomenhan L.C., Pamplona B.C., da Silva C.B., Marcucci M.C., Mimica L.M. (2011). Evaluation of the in vitro antimicrobial activity of an ethanol extract of Brazilian classified propolis on strains of *Staphylococcus aureus*. Braz. J. Microbiol..

[26] Fernandes J.R.A., Sugizaki M.F., Fogo M.L., Funari S.R.C., Lopes C.A.M. (1995). In vitro activity of propolis against bacterial and yeast pathogens isolated from human infections. J. Venom. Anim. Toxins..

[27] Pepeljnjak S., Kosalec I. (2004). Galangin expresses bactericidal activity against multiple-resistant bacteria: MRSA, *Enterococcus* spp. and *Pseudomonas aeruginosa*. FEMS Microbiol. Lett..

[28] Salomão K., Dantas A.P., Borba C.M., Campos L.C., Machado D.G., Neto F.R.A., de Castro S.L. (2004). Chemical composition and microbicidal activity of extracts from Brazilian and Bulgarian propolis. Lett. Appl. Microbiol..

[29] Mirzoeva O.K., Grishanin R.N., Calder P.C. (1997). Antimicrobial action of propolis and some of its components: The effects on growth, membrane potential and motility of bacteria. Microbiol. Res..

[30] Duarte S., Rosalen P.L., Hayacibar M.F., Cury J.A., Bowen W.H., Marquis R.E., Rehder V.L., Sartoratto A., Ikegaki M., Koo H. (2006). The influence of a novel propolis on mutans streptococci biofilms and caries development in rats. Arch. Oral Biol..

[31] Savka M.A., Dailey L., Popova M., Mihaylova R., Merritt B., Masek M., Le P., Nor S.R., Ahmad M., Hudson A.O. (2015). Chemical composition and disruption of quorum sensing signaling in geographically diverse united states propolis. Evid. Based Complement. Altern. Med..

[32] Cushnie T.P.T., Hamilton V.E.S., Chapman D.G., Taylor P.W., Lamb A.J. (2007). Aggregation of *Staphylococcus aureus* following treatment with the antibacterial flavonol galangin. J. Appl. Microbiol..

[33] Ouyang J., Sun F., Feng W., Xie Y., Ren L., Chen Y. (2018). Antimicrobial activity of galangin and its effects on murein hydrolases of vancomycin-intermediate *Staphylococcus aureus* (VISA) strain Mu50. Chemotherapy.

[34] Cushnie T.P., Hamilton V.E., Lamb A.J. (2003). Assessment of the antibacterial activity of selected flavonoids and consideration of discrepancies between previous reports. Microbiol. Res..

[35] Jaisinghani R.N. (2017). Antibacterial properties of quercetin. Microbiol. Res..

[36] Soromou L.W., Zhang Y., Cui Y., Wei M., Chen N., Yang X., Huo M., Baldé A., Guan S., Deng X. (2013). Subinhibitory concentrations of pinocembrin exert anti-*Staphylococcus aureus* activity by reducingα-toxin expression. J. Appl. Microbiol..

[37] Biva I.J., Ndi C.P., Griesser H.J., Semple S.J. (2016). Antibacterial constituents of *Eremophila alternifolia*: An Australian aboriginal traditional medicinal plant. J. Ethnopharmacol..

[38] Nina N., Quispe C., Jiménez-Aspee F., Theoduloz C. (2015). Antibacterial activity, antioxidant effect and chemical composition of propolis from the Región del Maule, Central Chile. Molecules.

[39] Krol W., Scheller S., Shani J., Pietsz G., Czuba Z. (1993). Synergistic effect of ethanolic extract of propolis and antibiotics on the growth of *Staphylococcus aureus*. Arzneimittelforsch.

[40] Santos A., Bastos M., Uzeda M., Carvalho A. (2002). Antibacterial activity of Brazilian propolis and fractions against oral anaerobic bacteria. J. Ethnopharmacol..

[41] Liu B., Pop M. (2009). ARDB-antibiotic resistance genes database. Nucleic Acids Res..

[42] Campoccia D., Montanaro L., Speziale P., Arciola C.R. (2010). Antibiotic-loaded biomaterials and the risks for the spread of antibiotic resistance following their prophylactic and therapeutic clinical use. Biomaterials.

[43] Arciola C.R., Radin L., Alvergna P., Cenni E., Pizzoferrato A. (1993). Heparin surface treatment of poly(methylmethacrylate) alters adhesion of a *Staphylococcus aureus* strain: Utility of bacterial fatty acid analysis. Biomaterials.

[44] Tiller J.C., Liao C.J., Lewis K., Klibanov A.M. (2001). Designing surfaces that kill bacteria on contact. Proc. Natl. Acad. Sci. USA.

[45] Bazaka K., Jacob M.V., Crawford R.J., Ivanova E.P. (2012). Efficient surface modification of biomaterial to prevent biofilm formation and the attachment of microorganisms. Appl. Microbiol. Biotechnol..

[46] Marchese A., Arciola C.R., Coppo E., Barbieri R., Barreca D., Chebaibi S., Sobarzo-Sánchez E., Nabavi S.F., Nabavi S.M., Daglia M. (2018). The natural plant compound carvacrol as an antimicrobial and anti-biofilm agent: Mechanisms, synergies and bio-inspired anti-infective materials. Biofouling.

[47] Russo N., Cassinelli C., Torre E., Morra M., Iviglia G. (2019). Improvement of the physical properties of guided bone regeneration membrane from porcine pericardium by polyphenols-rich pomace extract. Materials.

[48] Williams D.F. (2019). Challenges with the development of biomaterials for sustainable tissue engineering. Front. Bioeng. Biotechnol..

[49] Eskandarinia A., Kefayat A., Rafienia M., Agheb M., Navid S., Ebrahimpour K. (2019). Cornstarch-based wound dressing incorporated with hyaluronic acid and propolis: In vitro and in vivo studies. Carbohydr. Polym..

[50] Voss G.T., Gularte M.S., Vogt A.G., Giongo J.L., Vaucher R.A., Echenique J.V.Z., Soares M.P., Luchese C., Wilhelm E.A., Fajardo A.R. (2018). Polysaccharide-based film loaded with vitamin C and propolis: A promising device to accelerate diabetic wound healing. Int. J. Pharm..

[51] Oliveira R.N., McGuinness G.B., Rouze R., Quilty B., Cahill P., Soares G.D.A., Thiré R.M.S.M. (2015). PVA hydrogels loaded with a Brazilian propolis for burn wound healing applications. J. Appl. Polym. Sci..

[52] Boni B.O.O., Lamboni L., Souho T., Gauthier M., Yang G. (2019). Immunomodulation and cellular response to biomaterials: The overriding role of neutrophils in healing. Mater. Horiz..

[53] Kmiotek M., Bielinski D., Piotrowska M. (2018). Propolis as an antidegradant and biocidal agent for natural rubber. J. Appl. Polym. Sci..

[54] Ong T.H., Chitra E., Ramamurthy S., Ling C.C.S., Ambu S.P., Davamani F. (2019). Cationic chitosan-propolis nanoparticles alter the zeta potential of *S. epidermidis*, inhibit biofilm formation by modulating gene expression and exhibit synergism with antibiotics. PLoS ONE.

[55] Kim J.I., Pant H.R., Sim H.J., Lee K.M., Kim C.S. (2014). Electrospun propolis/polyurethane composite nanofibers for biomedical applications. Mater. Sci. Eng. C Mater. Biol. Appl..

[56] Patra J.K., Das G., Fraceto L.F., Campos E.V.R., Rodriguez-Torres M.D.P., Acosta-Torres L.S., Diaz-Torres L.A., Grillo R., Swamy M.K., Sharma S. (2018). Nano based drug delivery systems: Recent developments and future prospects. J. Nanobiotechnol.

[57] Reinholz J., Landfester K., Mailänder V. (2018). The challenges of oral drug delivery via nanocarriers. Drug Deliv..

[58] Arafa M.G., Ghalwash D., El-Kersh D.M., Elmazar M.M. (2018). Propolis-based niosomes as oromuco-adhesive films: A randomized clinical trial of a therapeutic drug delivery platform for the treatment of oral recurrent aphthous ulcers. Sci. Rep..

[59] Chen C.N., Weng M.S., Wu C.L., Lin J.K. (2004). Comparison of radical scavenging activity, cytotoxic effects and apoptosis induction in human melanoma cells by Taiwanese propolis from different sources. Evid. Based Complement. Altern. Med..

[60] Sadeghi-Aliabadi H., Hamzeh J., Mirian M. (2015). Investigation of Astragalus honey and propolis extract’s cytotoxic effect on two human cancer cell lines and their oncogen and proapoptotic gene expression profiles. Adv. Biomed. Res..

